# Weakness After an Intra-articular Steroid Injection: A Case Report of Acute Steroid-induced Myopathy

**DOI:** 10.5811/cpcem.2022.2.55995

**Published:** 2022-04-25

**Authors:** Matthew R. Jordan, Lauren A. Hensley, Michael L. Jackson

**Affiliations:** * Navy Medicine Readiness and Training Command, Department of Emergency Medicine, Camp Lejeune, North Carolina; † Navy Medicine Readiness and Training Command, Department of Internal Medicine, Portsmouth, Virginia; ‡ Navy Medicine Readiness and Training Command, Department of Emergency Medicine, Portsmouth, Virginia

**Keywords:** myopathy, steroid, glucocorticoids, intraarticular, case report

## Abstract

**Introduction:**

Weakness is a common chief complaint in the emergency department, and the use of glucocorticoids is pervasive in medicine. Muscle weakness, or myopathy, is a well documented side effect of chronic glucocorticoid use. However, acute myopathy, with an onset shortly after initiation of glucocorticoids, is much rarer.

**Case Report:**

We present a case of acute steroid-induced myopathy after a single intra-articular dose of triamcinolone in a young, healthy, active male. To our knowledge, this is the first case described in the medical literature of acute steroid-induced myopathy following a single intra-articular injection.

**Conclusion:**

In a patient who presents with proximal muscle weakness and has a history of glucocorticoid use, the diagnosis of steroid-induced myopathy should be considered. Acute steroid-induced myopathy should be high on the differential in a patient who presents with typical symptoms and has been prescribed glucocorticoids for less than 14 days or, in rare cases, may have recently received a single dose of glucocorticoids. Treatment is supportive and outpatient management is typically indicated, as respiratory muscle involvement is rare.

## INTRODUCTION

The differential diagnosis of muscle weakness, or myopathy, is very wide. The use of glucocorticoids has been associated with myopathy, typically occurring in patients with long-term oral steroid use. Acute steroid-induced myopathy, developing within 14 days of initiation of glucocorticoids in ambulatory patients, is poorly recognized and rare, with less than 20 cases documented in the literature.^1^ This case report details a presentation of acute steroid-induced myopathy. To our knowledge, it is the first case described in the medical literature following a one-time intra-articular injection.

## CASE REPORT

A 39-year-old male with a history of osteoarthritis, on as-needed non-steroidal anti-inflammatories, presented to the emergency department (ED) around 3 am with acute onset of bilateral lower extremity proximal muscle weakness. The patient reported going to bed that evening without any issue and awoke around 2 am to go to the bathroom, which was not unusual for him. However, he noticed profound weakness of the lower extremities. He could not flex at the hips and was unable to swing his legs out of bed. Eventually the patient was able to pull himself out of bed and slowly guide himself to the bathroom, noting continued severe weakness in his lower extremities. He also noted lacking the usual shoulder strength to wipe himself after using the bathroom. Approximately 18 hours prior to presentation to the ED, he had undergone a fluoroscopy-guided, intra-articular steroid injection of his right shoulder. The injection consisted of 7 milliliters (mL) of triamcinolone/ropivacaine mixture containing 2 mL of triamcinolone 40 milligrams per milliliter (mg/mL) (total of 80 mg of triamcinolone) and 5 mL of ropivacaine 0.75%. The procedure was uncomplicated, and he reported a 1/10 pain level post-procedure, down from 5/10 pre-procedure.

Upon presentation to the ED, he was well appearing, well nourished, and in no acute distress. He was afebrile and vital signs were as follows: heart rate 110 beats per minute; blood pressure 140/80 millimeters of mercury (mm Hg); respiratory rate 20 breaths per minute; oxygen saturation 98%; and temperature 97.5°F. Neurologic exam revealed 5/5 strength in his bilateral upper extremities, as well as at the knees and ankles bilaterally. However, he possessed only 3+/5 flexion strength of the bilateral hips. Patellar and Achilles reflexes were 2+ bilaterally. Vibratory sense was intact in both feet. Rectal tone was normal. The rest of his physical exam was unremarkable.

A differential diagnosis was formulated to include stroke, spinal compression syndrome, Guillain-Barré syndrome, rhabdomyolysis, electrolyte derangement, including hypokalemic periodic paralysis, transverse myelitis, myositis, and myopathy.

Emergency department diagnostics included a complete blood count, complete metabolic panel, creatine kinase (CK), erythrocyte sedimentation rate, and C-reactive protein level, all of which were within the reference ranges. Peak flow was 500 liters per minute (L/min) (normal range 300–660 L/min), and post-void residual volume on point-of-care ultrasound was 70 mL (normal <200 mL). Urinalysis showed greater than 500 mg/dL glucose (reference range negative), trace ketones (reference range negative), and small protein (reference range negative). Given the patient’s history of glucocorticoid use, exam with decreased hip flexion strength, reassuring lab work, peak flow, and post-void residual, a diagnosis of acute steroid-induced myopathy was made.

The internal medicine team was consulted to evaluate the patient in the ED. They agreed with the diagnosis of acute steroid-induced myopathy. The patient reported some improvement in his strength while in the ED, although not back to baseline, but he was able to ambulate. Neurology was consulted by the internal medicine team. Given his improvement in the ED, it was recommended by neurology that the patient follow up as an outpatient. The patient was discharged from the ED and followed up in the neurology clinic three days later. At that time, he reported complete resolution of his symptoms about 48 hours after presentation to the ED without residual deficits. Neurology ultimately diagnosed him with a transient myopathy and recommended that he avoid intra-articular glucocorticoid injections in the future.

**CPC-EM Capsule**

What do we already know about this clinical entity?

*Weakness, or myopathy, is a well documented side effect of chronic glucocorticoid use. Myopathy in the acute setting of glucocorticoid use is much rarer*.

What makes this presentation of disease reportable?

*This case report describes the first case in the medical literature of acute steroid-induced myopathy after a single intra-articular injection*.

What is the major learning point?

*In a patient with muscle weakness who has been on glucocorticoids, either single dose or long term, the diagnosis of steroid-induced myopathy must be considered*.

How might this improve emergency medicine practice?

*The diagnosis should be considered in the correct clinical picture. Myopathy should also be considered as a potential side effect of short-dose glucocorticoid use*.

## DISCUSSION

Weakness is a common presenting symptom in the ED. Due to its vague nature, it encompasses complaints ranging from generalized malaise to focal motor weakness and in severity from routine to emergent. This case details a presentation of weakness ultimately diagnosed as acute steroid-induced myopathy. This is a rare syndrome characterized by development of muscle weakness, most commonly affecting the proximal large muscle groups, within 14 days of initiation of glucocorticoids. Pelvic girdle and proximal lower extremity muscles are typically more severely involved than the upper extremities with sparing of the cranial nerves and sphincters. Rarely, the respiratory and/or distal muscles can be affected.^1^,^2^ Acute steroid-induced myopathy is rarer than a myopathy developing in patients on long-term glucocorticoids or in those admitted to critical care units on high-dose intravenous (IV) glucocorticoids.

Acute steroid-induced myopathy is highly unpredictable and should be considered in any patient with muscle weakness previously treated with glucocorticoids regardless of dose, route of administration, or duration of treatment. A previous case report detailed two patients who developed symptoms within hours of steroid administration.^1^ This same case report also reviewed all known case reports of acute steroid-induced myopathy in the literature, totaling 16 cases. The routes of administration were oral (eight cases), IV (five cases), and intramuscular, epidural, and intranasal (each with one case). No cases were intra-articular. Dosing was highly variable with one patient developing symptoms after a single dose of 40 mg of oral prednisone, while another was administered 1000 mg of IV hydrocortisone for 10 days before developing symptoms. Time to myopathy onset was between one hour and four weeks. Most made a full recovery over weeks to months. A single case of triamcinolone-induced acute steroid myopathy from an epidural injection has also previously been reported.^3^

Symptoms tend to occur more often with the fluorinated glucocorticoids, such as dexamethasone, triamcinolone, and betamethasone.^4^ Serum levels of muscle-associated enzymes such as CK and lactate dehydrogenase are usually normal. Electromyography is also typically normal, especially in the early phase of disease. Muscle biopsy tends to be non-specific, making acute steroid-induced myopathy a clinical diagnosis.^1^,^2^ A high index of suspicion is required to make the diagnosis.

Clinical improvement is expected over weeks to months with cessation of steroids or changing to non-fluorinated glucocorticoids, such as prednisone. Treatment is otherwise supportive.

In the rare case of respiratory muscle involvement, the patient should be admitted to a monitored unit for close airway observation. Otherwise, ED disposition centers around the patient’s functional ability. A physical therapy and occupational therapy evaluation may be appropriate. If being discharged, patients should have close follow up with neurology. Additionally, the patient should follow up with the physician prescribing his glucocorticoids. This clinician will need to reevaluate the need for glucocorticoids with the patient and discuss risks of continuing. Patients presenting with weakness who are prescribed short-burst dose glucocorticoids or who present after a single dose of glucocorticoid should be counseled to avoid further doses, if feasible, pending completion of appropriate follow-up medical care.

## CONCLUSION

In a patient who has received glucocorticoids and presents with muscle weakness, the diagnosis of steroid-induced myopathy should be considered. Glucocorticoid use can be of any duration, from chronic use to even a single dose. The associated muscle weakness tends to affect the proximal muscles, especially in the lower extremities. Therefore, acute steroid-induced myopathy should be considered in the differential of a patient presenting with proximal muscle weakness within 14 days of initiation of glucocorticoids. To our knowledge, this is the first case in the literature of an onset of acute steroid-induced myopathy following a single intra-articular injection.

## References

[1] Haran M, Scattner A, Kozak N (2018). Acute steroid myopathy: a highly overlooked entity. QJM.

[2] Pereira RM, Freire de Carvalho J (2011). Glucocorticoid-induced myopathy. Joint Bone Spine.

[3] Boonen S, Van Distel G, Westhovers R (1995). Steroid myopathy induced by epidural triamcinolone injection. Br J Rheumatol.

[4] Gupta A, Gupta Y (2013). Glucocorticoid-induced myopathy: pathophysiology, diagnosis, and treatment. Indian J Endocrinol Metab.

